# Use of Long Spinal Board Post-Application of Protocol for Spinal Motion Restriction for Spinal Cord Injury

**DOI:** 10.5811/westjem.18342

**Published:** 2024-08-27

**Authors:** Amber D. Rice, Philipp L. Hannan, Memu-iye Kamara, Joshua B. Gaither, Robyn Blust, Vatsal Chikani, Franco Castro-Marin, Gail Bradley, Bentley J. Bobrow, Rachel Munn, Mary Knotts, Justin Lara

**Affiliations:** * College of Medicine, Department of Emergency Medicine, The University of Arizona, Tucson, Arizona; † College of Medicine, Department of Emergency Medicine, The University of Arizona, Phoenix, Arizona; ‡ HonorHealth Scottsdale Osborn Medical Center, Emergency Department, Scottsdale, Arizona; § University of Arizona, Arizona Emergency Medicine Research Center, College of Medicine, Tucson, Arizona; ∥ ADHS Bureau of EMS and Trauma, Arizona Department of Health Services, Phoenix, Arizona; ¶ McGovern Medical School, Texas Emergency Medicine Research Center, Houston, Texas; # McGovern Medical School at The University of Texas Health Science Center at Houston, Houston, Texas; ** Tower Health - Reading Hospital, Philadelphia College of Osteopathic Medicine, Reading, Pennsylvania

## Abstract

**Introduction:**

Historically, prehospital care of trauma patients has included nearly universal use of a cervical collar (C-collar) and long spine board (LSB). Due to recent evidence demonstrating harm in using LSBs, implementation of new spinal motion restriction (SMR) protocols in the prehospital setting should reduce LSB use, even among patients with spinal cord injury. Our goal in this study was to evaluate the rates of and reasons for LSB use in high-risk patients—those with hospital-diagnosed spinal cord injury (SCI)—after statewide implementation of SMR protocols.

**Methods:**

Applying data from a state emergency medical services (EMS) registry to a state hospital discharge database, we identified cases in which a participating EMS agency provided care for a patient later diagnosed in the hospital with a SCI. Cases were then retrospectively reviewed to determine the prevalence of both LSB and C-collar use before and after agency adoption of a SMR protocol. We reviewed cases with LSB use after SMR protocol implementation to determine the motivations driving continued LSB use. We used simple descriptive statistics, odds ratios (OR) with 95% confidence intervals (CI) to describe the results.

**Results:**

We identified 52 EMS agencies in the state of Arizona with 417,979 encounters. There were 225 patients with SCI, of whom 74 were excluded. The LSBs were used in 52 pre-SMR (81%) and 49 post-SMR (56%) cases. The odds of LSB use after SMR protocol implementation was 70% lower than it had been before implementation (OR 0.297, 95% CI 0.139–0.643; *P* = 0.002). Use of a C-collar after SMR implementation was not significantly changed (OR 0.51, 95% CI 0.23–1.143; *P* = 0.10). In the 49 cases of LSB use after agency SMR implementation, the most common reasons for LSB placement were ease of lifting (63%), placement by non-transporting agency (18%), and extrication (16.3%). High suspicion of SCI was determined as the primary or secondary reason for not removing LSB after assessment in 63% of those with LSB placement, followed by multiple transfers required (20%), and critical illness (10%).

**Conclusion:**

Implementation of selective spinal motion restriction protocols was associated with a statistically significant decrease in the utilization of long spine boards among prehospital patients with acute traumatic spinal cord injury.

Population Health Research Capsule What do we already know about this issue?

*Long spine boards (LSB) have been shown to cause harm. Spinal motion restriction (SMR) protocols aim to reduce LSB use in patients with suspected spinal injury to minimize negative effects.*

What was the research question?

*We sought to evaluate the rates of and reasons for LSB use in high-risk patients after statewide implementation of a SMR protocol.*

What was the major finding of the study?

*Statewide SMR protocol implementation was associated with a 70% lower rate in LSB use* *(OR 0.297, 95% CI 0.139–0.643;* *P = 0.002).*

How does this improve population health?

*Implementation of SMR protocols decreases LSB use and, thus, the potential harms resulting using them.*

## INTRODUCTION

Prehospital care of trauma patients in the United States has historically included the near universal use of spinal immobilization (SI) by prehospital professionals.^1^ Traditional SI includes cervical collar (C-collar) application, a long rigid spine board (LSB), securing straps, and head blocks or other rotational support.^1^ The historical rationale to maintain this practice assumes safety and efficacy of traditional SI and aims to minimize medicolegal concerns, high morbidity, and cost associated with spinal cord injuries (SCI).^2^ Assumptions have been made throughout the years that SI performed in this manner is protective by reducing movement of potential spinal fractures and that the risk of secondary SCI and associated morbidity is mitigated with use of LSB.^2^

Prehospital evidence to support these assumptions is sparse, leaving open to question the supposed benefits.^3^ ^–^ ^6^ Hauswald et al performed a large, retrospective review comparing immobilized and non-immobilized trauma patients and demonstrated lower rates of neurologic injury in patients who were not immobilized on LSBs.^7^ Further complicating the use of LSBs is the difficulty in quantifying the actual risks and benefits of SI due to the complex nature of SCI.^8^ ^–^ ^10^ Numerous studies describe the real and potential harms of using rigid LSBs and other traditional practices of SI equipment during the care of acutely injured patients. While LSB use may facilitate extrications in the trauma setting, their use increases morbidity by causing pain and injury, including pressure necrosis, especially in patients requiring long transport times.^11^ Furthermore, LSB use has been correlated with an increased number of radiology studies ordered and subsequent radiation exposure to patients, increased hospital cost, inhibition of respiratory function, and increased intracranial pressure.^11^ ^–^ ^18^

In 2018, a position statement on spinal motion restriction (SMR) was published by the American College of Emergency Physicians, the National Association of EMS Physicians, and the American College of Surgeons Committee on Trauma. This position statement highlighted the need for SMR as the preferred method of reducing spinal motion after an injury and that complete SI is not possible. This position statement helped provide a standard of care for prehospital patients with possible SCI, including language stating that LSBs “should not be used as a therapeutic intervention or precautionary measure.”^19^ To address this recommendation, the state of Arizona implemented a statewide SMR protocol with the goal of decreasing LSB use in high-risk trauma patients. This protocol (IMAGE) de-emphasized LSBs by recommending that patient time on LSBs be minimized.

In this study we aimed to evaluate rates of LSB use after statewide implementation of this SMR protocol in high-risk trauma patients, whom we defined as having a discharge diagnosis of SCI. This study will inform future research on the effectiveness of SMR protocol implementation and hopefully result in the reduction of secondary injury in patients with SCI. Additionally, we aimed to clarify clinical reasoning used by clinicians in their decision to apply and not later remove a LSB, in contrast to SMR protocol guidance.

## METHODS

### Data Sources and Study Population

This was a retrospective, observational, multiagency, prehospital study including cases from January 1, 2013– December 31, 2015 in the Arizona Prehospital Information and Emergency Medical Services Registry System (AZ-PIERS) and the Hospital Discharge Database (HDD). Both data sources are maintained by the Arizona Department of Health Services. The AZ-PIERS dataset, which is managed by the department’s Bureau of Emergency Medical Services and Trauma System, is a voluntary patient registry that allows EMS agencies to collect and transmit electronic patient care data to the State. The database includes both required and optional reporting elements in National EMS Information System format. The AZ-PIERS captures agency information, patient demographics, response times, incident location, and treatment. The HDD collects inpatient and emergency department visits from all Arizona licensed hospitals except federal healthcare facilities such as the Veteran’s Administration, Department of Defense, or tribal hospitals.

The EMS transports in AZ-PIERS were linked to the state discharge database using a stepwise deterministic linkage algorithm with direct identifiers (first name, last name, date of birth, Social Security number, gender, date of incident/hospital admission, hospital name). Pre- and post-SMR protocol implementation cohorts were identified based on agency protocol implementation date, excluding a three-month run-in period. For agencies that implemented an SMR protocol during the study period, we reviewed the protocol to verify that critical components of SMR were present. These components included the following: protocol application to patients with traumatic injury; identification of a subgroup of patients very unlikely to have a spinal injury who were subsequently excluded; and restriction of spinal motion without requiring LSB use, meaning that a C-collar, scoop stretcher, vacuum splint, or ambulance stretcher was used. Of note, SMR protocols did require the use of a C-collar and most allowed for the patient to be positioned with the head of the gurney elevated to 30° if the patient did not have pain with elevation for the head of the bed.

Of the cases with matched EMS and hospital data, this study included only patients with a hospital-diagnosed SCI. These patients were included because of the higher risk nature of injury and risk for subsequent secondary SCI. These patients were identified as those with a principal diagnosis of traumatic injury (International Classification of Diseases, 9^th^ and 10^th^ Revisions, Clinical Modification [ICD-9] code 800–959 or [ICD-10] code S00–T34 or T79) mapping into the US Centers for Disease Control and Prevention’s ICD-9-CM (Barell matrix) and the proposed framework for ICD-10-CM diagnosis codes for a diagnosis of SCI.^20^ For those cases identified, prehospital documentation was reviewed to determine C-collar and LSB use. Cases were excluded if agency SMR implementation date was unknown; encounters were noted to be in duplicate; patients had no trauma in the prior 24 hours; reviewers deemed insufficient documentation to determine method of immobilization; or management involved interfacility transport. The prevalence of both LSB and C-collar use was determined among patients with SCI and compared between the pre- and post-SMR cohorts using simple descriptive statistics including odds ratios (OR) with 95% confidence intervals (CI).

The cases of SCI post-SMR implementation that had LSB placed by prehospital crew were secondarily reviewed by two independent physician reviewers using a qualitative methodology to determine the likely reasons for LSB use. They categorized cases based on what they determined to be the most likely reason for applying a LSB and for not removing it prior to transport. We used simple descriptive statistics to analyze this qualitative analysis, with calculation of kappa statistic to evaluate the reliability of the two raters’ determination.

The following possible reasons for initial and continued LSB use were defined a priori:

• To improve the ease of lifting due to the location of the injured patient relative to the ambulance gurney or size of the patient.
• LSB was placed by a non-transporting agency on scene.
• LSB was required for extrication of the patient from a difficult-to-access location.
• Cases in which the reviewers were unable to determine the reason for placing the patient on a LSB.

The following possible reasons for not removing the LSB once in the transport vehicle were defined a priori:

• Documented neurologic symptoms or other documented finding making the patient high probability of having SCI.
• Patient required LSB for multiple transfers from one agency to another for transport purposes.
• Documented medically complicated patient with critical illness, who had altered level of consciousness or was intubated.
• Cases in which the reviewers were unable to determine a clear reason for maintaining LSB immobilization throughout transport.

### Human Subjects Committee Review

This study was reviewed by the Arizona Department of Health Services Human Subjects Review Board and approved for publication on March 17, 2016.

## RESULTS

**Figure 1.**
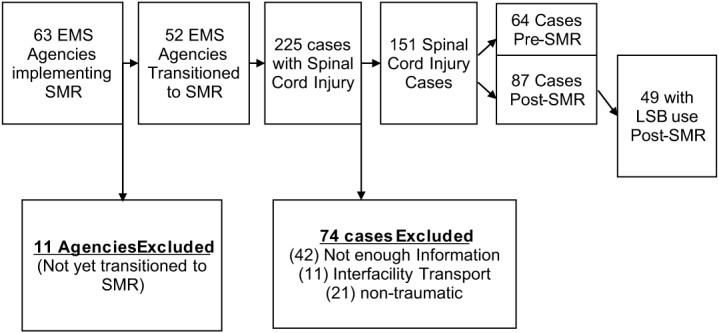
Flowchart of spinal cord injury cases and long spinal board use before and after implemention of a spinal motion restriction protocol. *EMS*, emergency medical services; *SMR*, spinal motion restriction.

There were 1,123,178 EMS transports entered into the AZ-PIERS dataset during the study period, and a total of 1,005,978 (89.6%) were successfully linked to HDD cases. We included 63 EMS agencies with a known SMR implementation status in the analysis. Of these, 52 transitioned to an SMR protocol, resulting in identification of 417,979 EMS encounters in the full study population. From those, we identified a cohort of patients with any diagnosis of spinal trauma, totaling 5,178 encounters. Within this population, 225 unique SCI cases were identified. Narrative reports of those records were examined by two independent reviewers to determine the method of immobilization. Seventy-four cases were excluded from the analysis for being SCI of nontraumatic cause (21), interfacility transfers (11), and those that did not contain enough information to determine the method of immobilization (42). The study group included 151 cases, which were divided into pre- (64 cases) and post-implementation (87 cases) cohorts. (Figure 1)

The distribution of demographic, mechanism/intent of injury, and outcome information were similar between the pre- and post-SMR cohorts as illustrated in Table 1. The results of the primary analysis can be seen in Table 2. Of the 151 SCI cases included, LSBs were used in 52 pre-SMR (81%) and 49 post-SMR (56%) cases. The odds of LSB use after SMR implementation were 70% lower than it was before implementation (OR 0.297, 95% CI 0.139–0.643; *P* = 0.002). C-collar use after SMR implementation was not significantly changed (OR = 0.51, 95% CI: 0.23–1.143; *P* = 0.10).

**Table 1.** Demographics.

	Pre-SMR	Post-SMR	*P*-value
	(N = 1,932)	(N = 3,246)	
Median age (Q1, Q3)	70 (48, 83)	70 (50, 84)	0.09
ISS, n (%)			
<15	1,631 (84.4%)	2,715 (83.6%)	0.46
> = 15	301 (15.6%)	531 (16.4%)	
Missing	0 (0.0%)	0 (0.0%)	
Gender, n (%)			
Female	1,072 (55.5%)	1,811 (55.8%)	0.83
Male	860 (44.5%)	1,435 (44.2%)	
Missing	0 (0.0%)	0 (0.0%)	
Race/ethnicity, n (%)			
White	1,557 (80.6%)	2,680 (82.6%)	<0.001
Black	28 (1.4%)	72 (2.2%)	
Hispanic/Latino	232 (12.0%)	373 (11.5%)	
Asian or Pacific Islander	22 (1.1%)	46 (1.4%)	
Native American or Alaskan Native	57 (3.0%)	44 (1.4%)	
Refused/unknown	36 (1.9%)	31 (1.0%)	
Mechanism of injury, n (%)			
Fall	1,015 (52.5%)	1,715 (52.8%)	0.25
Motor vehicle traffic	523 (27.1%)	874 (26.9%)	
Struck by/against	42 (2.2%)	67 (2.1%)	
Cut/pierce	1 (0.1%)	2 (0.1%)	
Overexertion	48 (2.5%)	49 (1.5%)	
Other	224 (11.6%)	351 (10.8%)	
Missing	79 (4.1%)	188 (5.8%)	
Intent of injury, n (%)			
Unintentional	1,855 (96.0%)	3,076 (94.8%)	0.91
Suicide	6 (0.3%)	10 (0.3%)	
Homicide	31 (1.6%)	49 (1.5%)	
Other	4 (0.2%)	4 (0.1%)	
Undetermined	36 (1.9%)	107 (3.3%)	
Hospital discharge status, n (%)			
Home	1,028 (53.2%)	1,609 (49.6%)	<0.001
SNF/ALF/rehab/long term	673 (34.8%)	1,312 (40.4%)	
Expired/hospice	68 (3.5%)	142 (4.4%)	
Other	163 (8.4%)	183 (5.6%)	

*ISS*, Injury Severity Score; *SNF*, skilled nursing facility; *ALF*, assisted living facility; *rehab*, rehabilitation facility.

**Table 2.** Rates of long spinal board and cervical-collar use pre- and post-implementation of a spinal motion restriction protocol.

	Pre-SMR implementation (n = 64)	Post-SMR implementation (n = 87)	Odds ratio (95% CI)
Patients with LSB placed for transport (%)	52 (81.25)	49 (56.32)	0.297 (0.139–0.643) *P* = 0.002
Patients with C-collar placed for transport (%)	53 (82.81)	62 (71.26)	0.51 (0.23–1.143) *P* = 0.10

*LSB*, long spinal board; *SMR*, spinal motion restriction; *CI*, confidence interval; *C-collar*, cervical collar.

The secondary analysis identifying reasons for LSB use after SMR protocol implementation are illustrated in Table 3. Of the 49 cases, the most common reasons for LSB placement were as follows: ease of lifting (63%); placement by non-transporting agency (18%); and extrication (16.3%). High suspicion of SCI was thought to be the primary or secondary reason for not removing LSB after assessment in the majority (53%) of cases, followed by multiple transfers required (10%), and critical illness (10%). In 26% of cases, there was not a clear reason for maintaining full spinal precautions throughout transport. There was a strong level of agreement between the raters’ determinations of the reasons for LSB placement and reasons for no discontinuation (kappa, 0.8209 and 0.8108, respectively).

**Table 3.** Reasons for long spinal board use in patients with diagnosed spinal cord injury.

Reason for LSB initial placement (n = 49)		Primary reason LSB was not removed prior to transport (n = 49)	
Extrication (%)	8 (16.33)	High suspicion of spinal cord injury (%)	26 (53.06)
Ease of lifting non-ambulatory patient from the ground (%)	31 (63.27)	Required transfer between agencies (%)	5 (10.20)
Placed by non-transporting agency (%)	9 (18.37)	Critical illness (ie, unconscious, intubated) (%)	5 (10.20)
Other (%)	1 (2.04)	Other or unclear reasoning (%)	13 (26.53)

*LSB*, long spinal board.

## DISCUSSION

Results of this study suggest that implementation of a selective SMR protocol, which focused on reducing LSB use, was associated with a statistically significant decrease in but not elimination of LSB use among prehospital patients with acute traumatic SCI. It is notable that there was such a significant decrease in LSB use even in the very highest risk cohort of trauma patients studied here—those with hospital-diagnosed SCI. As expected, the rate of C-collar use was not affected by these protocol changes as the protocol implemented did not make them optional. This data supports that the adoption of SMR protocols by prehospital agencies does lead to decreased rates of LSB use, even in patients with high-risk injuries, and may subsequently reduce the secondary comorbidity associated with these devices.

The use of C-collars did not decline significantly as a result of the protocol changes, as EMS professionals were required to use the device for patients meeting high-risk criteria. This finding was expected given that the SMR protocol requires continued use of these tools to limit spinal motion. The observation that rates of C-collar use did not decrease lends additional support to the conclusion that the SMR protocol resulted in a decrease in LSB use rather than other system-related changes or confounders resulting in this change.

The most practical and least controversial use of LSB in trauma care is for extrication. It does seem that this was a factor in a small number of cases. Similarly, the most common reason for LSB use after SMR protocol implementation (63% of cases where LSBs were used) was that EMS professionals appeared to use the LSB to lift a non-ambulatory patient from the ground to the gurney. Perhaps more controversial is limiting the use of LSB for patients with exam findings suggestive of SCI. In this study, in more than half of cases with LSB placement, clinicians documented a concerning or abnormal neurological exam finding and documented that a LSB had been used due to a possible SCI. In these cases, perhaps improved educational outreach would limit LSB use to the practical need to lift a patient and encourage LSB removal prior to transportation.

In patients who had a LSB placed for their movement to a gurney, frequently no reason was cited as to why the patient was not rolled off the spine board prior to transportation. In some cases, the patient’s care required multiple transfers between agencies, or the patient was intubated or had other signs of critical illness or airway compromise, which may have made removing the spine board challenging or simply was not a priority in treatment at the time. 

## LIMITATIONS

Limitations of this study include the retrospective collection of the data from prehospital EMS documentation. Additionally, the AZ-PIERS database lacks data fields that would have provided specific reasons EMS used a LSB, and limited information was available from the narratives to qualitatively assess why they chose to use a LSB. As mentioned above, the reasons for LSB use were not clear in some of the narratives, and while providing insight into the thought process of the EMS professionals, the narratives may not represent the actual primary motivation for placing and not removing patients from LSBs. Neither were we able to guarantee that each EMS agency implemented their SMR protocol in the same manner. While many of the EMS agencies’ selective SMR protocols were reviewed for the presence of certain critical elements, they were not identical; and although standardized educational material was available, the uniformity of the education given to the the EMS professionals could not be evaluated.

## CONCLUSION

Prehospital use of a long spinal board in high-risk patients and those with a hospital-diagnosed spinal cord injury, significantly decreased after implementation of spinal motion restriction protocols. Continued use of the long spinal board after SMR protocol implementation appeared to be most common when EMS professionals perceived a practical difficulty or unease with being able to lift, move, and carry injured patients without the use of a LSB. As with all paradigms shifts in policy, it does take time for complete adherence to new practices and procedures. This is where visual feedback and quality improvement programs play a large role, highlighting the need to provide guidance regarding when a LSB should be used and the optimal timing of removing patients from a LSB to minimize complications.
